# Housing Affordability as a Social Determinant of Mental Health: Longitudinal Evidence from China

**DOI:** 10.3390/healthcare14070956

**Published:** 2026-04-06

**Authors:** Yi Chen, Chunqi Wu, Jianping Ye

**Affiliations:** 1 Department of Land & Real Estate Management, School of Public Administration and Policy, Renmin University of China, Beijing 100872, China; chenyi0527@ruc.edu.cn (Y.C.); jpye@ruc.edu.cn (J.Y.); 2 Institute of Urbanization, Beijing City University, Beijing 100083, China

**Keywords:** housing affordability, mental health, longitudinal study, social determinant of health

## Abstract

**Background**: Housing affordability is one of the most pressing social and political challenges in urban China, yet empirical evidence on its impact on residents’ mental health remains limited. **Methods**: Guided by the Social Determinants of Health (SDOH) framework, this study examines its relationship with mental health using nationally representative longitudinal data from the China Family Panel Studies (CFPS) across the 2016, 2018, and 2020 waves. We employ two-way fixed effects models and further incorporate an instrumental variable strategy to address potential endogeneity. **Results**: There is a significant association between housing affordability and mental health; greater affordability is associated with a lower likelihood of experiencing depressive symptoms. Heterogeneity analyses further reveal that these benefits vary by housing asset status and educational attainment. **Conclusions**: From an SDOH perspective, this study provides empirical evidence on how housing affordability contributes to mental health inequities in the Chinese context. Housing affordability should be recognized as a public health concern requiring coordinated policy responses. Targeted interventions are necessary to protect vulnerable populations that are most exposed to affordability shocks.

## 1. Introduction

Over the past few decades, there has been a marked and continuous decline in housing affordability in both developed and developing economies, making it one of the most pressing social and policy challenges worldwide [1,2,3,4]. Such affordability pressures have disproportionately affected younger generations and fueled widespread public narratives about the unattainability of homeownership. But beyond the access to homeownership per se, well-documented studies have highlighted that housing, as a social determinant, plays a central role in shaping health outcomes [5,6,7]. Recent studies have brought attention to the relationship between housing (un)affordability and its effects on mental health.

A growing body of literature shows a clear overall increase in the experience of unaffordable housing and a clear negative link between unaffordable housing and mental health consequences including anxiety, depression and behavioral problems [8,9,10]. However, there is still a lack of evidence investigating how associations are changing in the face of welfare and housing system reforms that have exacerbated insecurity and reduced support opportunities [11]. Understanding how housing affordability influences mental health across different contexts is critical for assessing social risks and population well-being in contemporary societies.

China provides a theoretically distinctive context for examining how housing operates as a key social determinant in shaping health outcomes. Firstly, since the market-oriented housing reform initiative in 1998, China has experienced more than two decades of rapid housing price growth, during which housing has shifted from a welfare-based provision to a highly marketized and increasingly financialized asset. As a result, housing affordability pressures have become particularly acute in urban areas [12]. Secondly, homeownership is deeply embedded in social norms and institutional arrangements related to marriage, family formation, and intergenerational responsibility [13]. A substantial body of research indicates that in urban China, homeownership is widely regarded as a prerequisite for marriage and for the completion of the transition to adulthood. The normative expectation of “marital housing” has profoundly shaped individuals’ housing choices, family decisions, and social identities [14,15]. Within this context, housing unaffordability does not merely increase economic pressure; it is also experienced as blocked life-course progression, unmet social expectations, and heightened downward social comparison.

Among various measures of housing affordability, the price-to-income ratio (PIR) has the longest history and the widest recognition, with the World Bank suggesting that a PIR in the range of 3–6 is generally considered affordable [12,14]. In China, however, housing prices rose by more than 10% annually in real terms between 2003 and 2014 [15], and the average PIR across 35 large and medium-sized cities increased from 5.38 in 1998 to 10.67 in 2018 [16]. The situation is even more severe in megacities such as Beijing and Shanghai. In 2018, Beijing’s PIR reached 48.13, ranking first among global cities and far exceeding that of London (12.19) and New York (12.34) [17]. Evidence based on PIR indicates that housing affordability in major Chinese cities has long deviated substantially from internationally recognized thresholds and has reached extreme levels in first-tier cities and megacities [12,14].

At the micro level, housing unaffordability adversely affects health and quality of life by generating financial stress, forcing trade-offs in essential expenditures, exposing households to substandard housing conditions, and constraining access to high-opportunity neighborhoods and schools, thereby reinforcing intergenerational disadvantage [18,19,20,21,22,23]. At the macro level, housing unaffordability may delay family formation, reduce fertility, and induce demographic shifts. It can also displace high-skilled labor, reduce allocative efficiency in labor markets, generate spatial mismatch, and ultimately undermine productivity, posing potential risks to macroeconomic stability and broader social well-being [24,25].

Within the Social Determinants of Health (SDOH) framework, housing is conceptualized as a direct determinant of health rather than merely an outcome of socioeconomic status (SES). Housing operates as a key pathway through which health inequities are produced, shaping mental health and other health outcomes through multiple mechanisms, including housing affordability, residential stability, housing conditions and quality, and neighborhood environments [26,27,28]. The SDOH framework further emphasizes that health disparities arise from broader structural inequities in social and economic conditions, including, but not limited to, the allocation of housing resources, thereby providing a useful lens for understanding housing affordability as embedded within wider patterns of social stratification and health inequity.

Although the literature on housing affordability and its mental health implications has grown considerably, empirical evidence from the Chinese context remains limited. This study contributes to the housing–health literature in three ways. First, leveraging three waves of nationally representative longitudinal data, we examine the effect of housing affordability on mental health, providing robust evidence within China. Second, we employ two-way fixed-effects regression and instrumental variable (IV) strategies to address endogeneity, ensuring rigorous empirical analysis. Third, our heterogeneity analysis identifies single-property homeowners as one of the most vulnerable groups. This finding, which differs markedly from results reported in prior studies, provides a meaningful extension to the existing housing–health literature. Collectively, these findings underscore the need for targeted housing support policies to enhance affordability, reduce health disparities, and promote broader social well-being.

The remainder of this paper is structured as follows: Section 2 details the data, variables, and methodology; Section 3 presents the empirical results; Section 4 offers a discussion; and Section 5 concludes with policy implications.

## 2. Materials and Methods

### 2.1. Data

This study utilizes data from the China Family Panel Studies (CFPS), a nationally representative longitudinal survey administered by Peking University. The CFPS employs multi-stage probability sampling, covering approximately 16,000 households across 25 provinces and municipalities through face-to-face interviews, telephone surveys, and self-completion questionnaires.

We analyze three waves of CFPS data (2016, 2018, and 2020) to construct our key variables. The survey data are supplemented with housing price information from the www.58.com real estate database and provincial macroeconomic indicators from the National Bureau of Statistics of China.

The CFPS achieves response rates comparable to international standards, with cross-sectional response rates of 62% (2020) and 69.3% (2018), and cross-wave retention rates of 77% and 86.6%, respectively. These rates are consistent with established international panels, ensuring data quality for rigorous empirical analysis.

Our final sample of 25,195 observations is based on the following criteria:

- Age restriction: 25–64 years (working-age population).
- Outlier exclusion: income <1000 RMB, household size >10 members, top 5% income.

### 2.2. Variables

#### 2.2.1. Dependent Variable

Mental Health. To measure psychological well-being, this study constructs a mental health index using the CES-D (Center for Epidemiologic Studies Depression Scale) items included in the CFPS (Table 1). The CES-D is a globally recognized and validated instrument for assessing depressive symptoms and mental health status. In our analytical sample, Cronbach’s α for the 8-item CES-D scale is 0.79, indicating satisfactory internal consistency reliability.

Respondents reported the frequency of specific symptoms over the past week using a four-point Likert scale. Although the original CFPS questionnaire utilized a 0–3 scoring format, these responses were re-scaled to a 1–4 range for this analysis to ensure consistency. Following standard CES-D scoring procedures, all eight items were summed to generate a composite mental health score. Higher scores indicate poorer mental health and greater depressive symptomatology. Specifically, items 4 (“I feel happy”) and 6 (“I have a happy life”) are positively worded; these were reverse-coded prior to summation to ensure that higher total scores consistently reflect poorer mental health.

#### 2.2.2. Independent Variable

Housing affordability. We measure housing affordability using the price-to-income ratio (PIR), defined as the average commercial housing price per square meter in each respondent’s province divided by their monthly household income (in Chinese Yuan). For ease of interpretation, we operationalize affordability as the inverse of the PIR, so that higher values indicate greater affordability.

Provincial-level housing price data are obtained from the National Bureau of Statistics for the years 2016, 2018, and 2020. Monthly household income is derived from self-reported responses in the CFPS adult questionnaire, converted to comparable units for ratio calculation.

#### 2.2.3. Control Variable

To mitigate omitted variable bias, this study controls for a series of potential confounders that may simultaneously affect housing affordability and mental health, with these variables categorized at multi-levels to align with the Social Determinants of Health (SDOH) framework. First, individual-level demographic characteristics (e.g., age, gender, marital status, and presence of children) are included to capture fundamental disparities in status. Second, household-level socioeconomic variables, such as education, employment status and total household assets, are incorporated to reflect household economic resilience. Finally, we account for regional-level factors, specifically provincial GDP per capita and population density, to characterize the macroeconomic environment. These variables are widely adopted in the literature concerning the housing-health nexus, aiding in the mitigation of potential omitted variable bias.

### 2.3. Methods

To examine the effect of housing affordability on mental health, we estimate two-way fixed effects models with province and year fixed effects. Province fixed effects absorb time-invariant provincial heterogeneity, while year fixed effects control for common temporal shocks.

The baseline model is specified as follows:

$${Mental\_Health}_{ict}=\beta_{0}+\beta_{1}{Affordability}_{ict}+\gamma X_{ict}+\delta_{t}+\eta_{c}+\varepsilon_{ict}$$

In this model, ${Mental\_Health}_{ict}\;$ denotes the mental health status of individual *i* in region c in year t; ${Affordability}_{ict}$ represents the housing affordability of individual *i* in region c at time t; $X_{ict}$ is a vector of control variables. $\delta_{t}$ captures time fixed effects. $\eta_{c}$ represents provincial fixed effects. $\varepsilon_{ict}$ is the error term. Standard errors are clustered at the provincial level to account for potential correlation of unobserved factors within provinces. All analyses were performed using Stata version 16.0.

## 3. Results

### 3.1. Descriptive Analysis

Table 2 presents the descriptive statistics for the analytical sample. The mean mental health score is 13.515 (where higher scores indicate poorer mental health), suggesting a moderate level of overall psychological distress within the sample.

### 3.2. Baseline Regression Results

Prior to regression analysis, we assessed multicollinearity using variance inflation factors (VIFs). All VIF values were consistently below the threshold of 10, indicating the absence of significant multicollinearity issues.

Table 3 presents the two-way fixed effects regression results. Given that higher mental health scores indicate poorer psychological well-being, the negative coefficients suggest that greater housing affordability is associated with better mental health. In the baseline model (Model 1), the coefficient for housing affordability is −0.716 (*p* < 0.01). After including individual- and household-level covariates in Model 2, the coefficient remains significant at −0.543 (*p* < 0.01), suggesting that the association is robust to the inclusion of these controls.

In Model 2, being married, employed, and having higher education is associated with better mental health. These findings are consistent with previous research [20].

### 3.3. Robustness Checks

#### 3.3.1. Instrumental Variable Strategy

To address potential endogeneity, such as omitted variable bias and reverse causality, we implement an instrumental variable (IV) approach, using the logarithm of land transfer prices under China’s “bidding, auction, and listing” (*zhaopaigua*) system as an instrument for housing affordability. Land prices are strongly related to housing costs but are unlikely to directly affect individual mental health, meeting the relevance and exclusion restrictions.

We estimate a two-stage least squares (2SLS) model with provincial-clustered standard errors. These results are presented in Table 4. The first stage shows a strong correlation between land prices and affordability (coefficient = −0.149, *p* < 0.01), with a first-stage F-statistic of 484.341, well above the conventional threshold of 10, confirming instrument strength. In the second stage, the instrumented affordability coefficient is −0.680 (*p* < 0.01), which is larger in magnitude than the baseline fixed-effects estimate (−0.543), suggesting that the original fixed-effects estimate may be biased toward zero.

#### 3.3.2. Alternative Outcome Measures

To evaluate the robustness of our baseline findings, we also adopt the strategy of using alternative outcome measures. Specifically, we replace the original mental health outcome with two other measures of subjective well-being: happiness and life satisfaction. *Happiness* and *life satisfaction* were sourced from self-reported CFPS data. Happiness was measured on a 0–10 scale, while life satisfaction used a 1–5 scale (“very satisfied” = 5). As higher scores on the main dependent variable reflect greater psychological distress, we reverse-coded these measures to maintain directional consistency. Consequently, in our robustness checks, a negative coefficient consistently indicates that improved housing affordability enhances subjective well-being. The robustness checks (Table 5) confirm the stability of our main findings across different specifications. When using these alternative outcomes, the results align with the conceptual direction of improved mental health, consistent with our baseline findings.

#### 3.3.3. Sample Restrictions

For sample restrictions, we exclude individuals without local household registration (hukou), i.e., migrant workers who were not born in the city where they were interviewed. This is because residents with hukou have different rights regarding housing eligibility in metropolitan cities, as well as access to education, healthcare, and social security benefits. When restricting the sample to respondents with hukou, housing affordability remains significantly associated with better well-being (coefficient = −0.542, *p* < 0.01), suggesting that our results are robust to sample selection (Table 5).

These robustness checks indicate that improved housing affordability consistently enhances psychological well-being across alternative outcome measures and sample restrictions, thereby reinforcing the credibility of our primary conclusion.

### 3.4. Heterogeneity

#### 3.4.1. Heterogeneity Analysis by Housing Asset Status

Recent studies have further examined how housing affordability interacts with ownership status in affecting mental health outcomes [8,10]. To conduct the heterogeneity analysis, we consider the distribution of urban homeownership in China, where 80–90% of households own a home, and over 20% own multiple residential properties [29,30]. Different housing asset statuses reflect distinct socio-economic positions. Accordingly, we stratify the sample into three groups based on housing asset status: renters, single-property homeowners, and multi-property homeowners.

Results in Table 6 indicate that housing affordability has a significant positive impact on mental health, with the most pronounced effect observed among single-property owners. This suggests that individuals who may face mortgage or other housing-related financial obligations could be particularly sensitive to changes in housing affordability.

#### 3.4.2. Heterogeneity Analysis by Educational Attainment

We also stratify by education, as it systematically identifies subgroups with distinct health and economic profiles. While individual responses vary randomly, aggregation reveals consistent differences across educational groups. Moreover, education can shape how individuals perceive and cope with housing-related financial stressors [31]. We stratify the sample into three groups: (1) junior high or below, (2) high school level, and (3) college degree or above.

Table 7 indicates that the mental health benefits of better housing affordability are most pronounced for lower-educated individuals. For those with junior high or below, improved affordability significantly reduces mental health stress (−0.623, *p* < 0.01), with a smaller but still significant effect for high school graduates (−0.560, *p* < 0.01). In contrast, housing affordability has no significant impact on college-educated individuals (0.042, *p* > 0.10). These findings highlight that lower educational attainment increases vulnerability to housing cost burdens, while higher-educated groups are relatively less affected.

## 4. Discussion

Drawing on nationally representative panel data from the China Family Panel Studies (2016–2020), this study systematically examines the relationship between housing affordability and mental health within a Social Determinants of Health (SDOH) framework. The results indicate that higher housing affordability is significantly associated with better mental health, and this association remains robust after controlling for a wide range of individual-, household-, and regional-level factors. These findings are highly consistent with existing international evidence on the relationship between housing affordability and mental health [20,32,33]. Beyond reaffirming the importance of housing costs in shaping mental health outcomes, our results suggest that housing affordability should not be understood merely as a reflection of individuals’ socioeconomic status, but rather as a key health determinant embedded in broader social structures and institutional contexts.

More importantly, to our knowledge, the pattern of vulnerability identified in the heterogeneity analysis differs markedly from that reported in prior studies. Existing studies typically find that private renters face greater mental health risks under housing unaffordability, while the effects among mortgaged homeowners are relatively weaker [10,11,32]. In contrast, our findings reveal that in the urban China context, the group most sensitive to housing affordability pressures is not renters, but households owning a single property. This result provides a meaningful extension to current housing–health literature.

In the Chinese context, homeownership carries strong normative significance that extends well beyond residential needs and is deeply embedded in marriage, family formation, and intergenerational responsibility [34]. The heightened vulnerability of single-property owners can be largely understood within the context of China’s exceptionally high urban homeownership rate, which has reached approximately 80–90%, compared with 45–60% in major cities of developed countries [29,35]. On the one hand, households owning a single dwelling often bear a relatively high mortgage burden, with housing expenditures exerting substantial crowding-out effects on household income. On the other hand, the lack of secondary housing assets as a buffer makes this group more susceptible to sustained financial stress in the face of housing price fluctuations, income instability, or macroeconomic shocks. This structural condition, characterized by high ownership but limited buffering capacity, renders housing affordability a salient source of psychological stress. It helps explain why, in an institutional context marked by widespread homeownership, owning a single dwelling does not necessarily ensure economic security; rather, it may be associated with elevated mental health risks.

Moreover, the heightened vulnerability observed among individuals with lower educational attainment further underscores the unequal effects of housing affordability within the processes of socioeconomic reproduction. Educational attainment shapes not only income potential and employment stability but also individuals’ perceptions of and responses to economic risk. Individuals with limited socioeconomic resources are more likely to accumulate persistent stress and insecurity in response to rising housing costs, thereby exacerbating mental health inequities [4,20,27]. Our findings are consistent with this theoretical expectation, indicating that housing affordability does not exert uniform effects across social groups.

## 5. Limitations

This study is subject to several limitations. First, the measurement of housing affordability remains a subject of methodological debate. In this study, we operationalize housing affordability using the price-to-income ratio based on provincial commercial housing prices and household income. The absence of detailed data on rental expenditures, utility costs, and the specific distinctions between homeowners and renters may reduce measurement precision and overlook the multidimensional nature of housing-related financial burdens.

Second, housing ownership is treated as a single category, whereas in China, private, market-based, and shared forms of ownership carry distinct economic burdens and wealth implications. These distinctions potentially affect health outcomes in complex ways, and the interactions between housing affordability and tenure types should be explored more thoroughly in future research.

Third, the limited scope of our three-wave panel restricts the ability to capture long-term transitions in housing affordability and their subsequent causal effects on mental health.

Additionally, we did not examine potential mediating mechanisms, such as deprivation and financial well-being, through which housing affordability shapes mental health outcomes.

All these areas require further investigation in the future.

## 6. Conclusions

This study provides empirical evidence from China demonstrating that housing affordability is significantly associated with mental health. This association remains robust even after fully adjusting for various individual-, household-, and regional-level covariates.

Specifically, individuals with greater housing affordability exhibit a lower likelihood of reporting depressive symptoms. Homeowners lacking sufficient asset buffers and individuals with limited socioeconomic resources are most vulnerable to housing affordability issues.

Although the cooling of the Chinese housing market since late 2021 has improved affordability, employment challenges amid China’s economic transition suggest that housing affordability will remain a critical policy priority. Concurrently, China faces population aging and declining birth rates, leading to a shrinking working-age population that may hinder long-term economic development.

The findings underscore that addressing housing affordability is essential not only for economic stability but also for reducing mental health inequalities. Consequently, housing affordability should be recognized as a public health concern requiring coordinated policy responses. Targeted interventions should prioritize disadvantaged populations who demonstrate greater vulnerability to socioeconomic shocks.

## Figures and Tables

**Table 1:** CFPS Survey Items for Mental Health Measurement.

Item	Frequency
1. I am in a low spirit.	1. Never (less than one day)
2. I find it difficult to do anything.	2. Sometimes (1–2 days)
3. I cannot sleep well.	3. Often (3–4 days)
4. I feel happy.	4. Most of the time (5–7 days)
5. I feel lonely.	
6. I have a happy life.	
7. I feel sad.	
8. I feel that I cannot continue with my life.	

**Table 2:** Summary Statistics for the Analytical Sample from CFPS 2016–2020 (*N* = 25,195).

	Mean	SD	Min	p50	Max
Mental Health	13.515	3.968	4	13	32
Affordability	1.266	0.636	0.198	1.134	5.103
Ln (age)	3.789	0.235	3.219	3.850	4.159
Gender (Male = 1)	0.497	0.500	0	0	1
Education	1.359	0.661	1	1	3
Married (=1)	0.917	0.276	0	1	1
Employed (=1)	0.895	0.307	0	1	1
Children (=1)	0.477	0.499	0	0	1
Total assets	0.509	1.125	−9.71	0.270	50.461
Ln (GDP per capita)	10.813	0.362	10.218	10.785	12.009
Population density	32.870	11.262	11.360	32.35	55.01

Notes: (1) Age is log-transformed; (2) Housing affordability is the inverse price-to-income ratio; (3) Education is coded 1–3; (4) Binary indicators denote marital status, employment, and the presence of children in the household; (5) Total assets are expressed in millions; provincial controls include Ln(GDP per capita) and scaled population density.

**Table 3:** Baseline Regression Results—Housing Affordability and Mental Health.

Variables	(1)	(2)
	Mental Health	Mental Health
Affordability	−0.716 ***	−0.543 ***
	(0.077)	(0.067)
Ln (age)		0.339 *
		(0.170)
Gender (Male = 1)		−0.869 ***
		(0.068)
Education		−0.233 ***
		(0.076)
Married (=1)		−1.578 ***
		(0.160)
Employed (=1)		−0.387 ***
		(0.114)
Children (=1)		0.032
		(0.062)
Total assets		−0.044
		(0.032)
Ln (GDP per capita)		−1.386
		(1.245)
Population density		−0.011
		(0.009)
Provincial FE	Yes	Yes
Year FE	Yes	Yes
Observations	25,195	25,195
R-squared	0.040	0.066

Note: *** *p* < 0.01, ** *p* < 0.05, * *p* < 0.1.

**Table 4:** Two-Stage Least Squares Estimation Results.

Variables	First Stage	Second Stage
	Affordability	Mental Health
Ln (Land price)	−0.149 ***	
	(0.003)	
Affordability (Instrumented)		−0.680 ***
		(0.074)
Ln (age)	−0.111 ***	0.292 *
	(0.017)	(0.170)
Gender (Male = 1)	0.002	−0.871 ***
	(0.003)	(0.069)
Education	0.059 ***	−0.201 **
	(0.009)	(0.076)
Married (=1)	0.063 ***	−1.542 ***
	(0.008)	(0.158)
Employed (=1)	−0.000	−0.384 ***
	(0.006)	(0.114)
Children (=1)	−0.032 ***	0.020
	(0.009)	(0.063)
Total assets	0.039 ***	−0.030
	(0.013)	(0.029)
Ln (GDP per capita)	−0.168 **	−1.410
	(0.075)	(1.244)
Population density	−0.003 ***	−0.012
	(0.001)	(0.009)
Fixed Effects		
Provincial FE	Yes	Yes
Year FE	Yes	Yes
Observations	25,195	25,195
R-squared	0.731	0.038
F-statistic	484.341	118.076

Note: *** *p* < 0.01, ** *p* < 0.05, * *p* < 0.1.

**Table 5:** Robustness Checks: Alternative Outcome Measures and Sample Restrictions.

Variables	Alternative Outcomes		Sample Restriction
	Happiness	Life Satisfaction	Hukou Sample
	(1)	(2)	(3)
Affordability	−2.644 ***	−1.505 ***	−0.542 ***
	(0.009)	(0.026)	(0.066)
Ln (age)	0.008	0.060 ***	0.337 *
	(0.016)	(0.015)	(0.170)
Gender (Male = 1)	0.003	−0.002	−0.868 ***
	(0.005)	(0.003)	(0.068)
Education	0.004	−0.011 *	−0.232 ***
	(0.008)	(0.005)	(0.076)
Married (=1)	0.000	−0.030 ***	−1.581 ***
	(0.018)	(0.009)	(0.160)
Employed (=1)	0.023 *	−0.003	−0.381 ***
	(0.012)	(0.008)	(0.112)
Children (=1)	−0.030 **	0.006	0.034
	(0.013)	(0.006)	(0.063)
Total assets	0.000	−0.004	−0.044
	(0.010)	(0.004)	(0.032)
Ln (GDP per capita)	−0.403 **	−0.017	−1.396
	(0.160)	(0.102)	(1.252)
Population density	0.004 **	0.001	−0.011
	(0.001)	(0.001)	(0.009)
Provincial FE	Yes	Yes	Yes
Year FE	Yes	Yes	Yes
Observations	25,195	25,195	25,181
R-squared	0.917	0.884	0.066

Note: *** *p* < 0.01, ** *p* < 0.05, * *p* < 0.1.

**Table 6:** Heterogeneity Effects by Housing Asset Status.

Variable	Renters	Single-Property Owners	Multiple-Property Owners
	Mental Health	Mental Health	Mental Health
Affordability	−0.311 **	−0.546 ***	−0.513 ***
	(0.117)	(0.079)	(0.109)
Ln (age)	−0.217	0.522 **	−0.042
	(0.469)	(0.242)	(0.471)
Gender (Male = 1)	−0.557 ***	−0.918 ***	−0.889 ***
	(0.178)	(0.074)	(0.123)
Education	−0.468 ***	−0.234 **	−0.085
	(0.120)	(0.086)	(0.094)
Married (=1)	−1.541 ***	−1.617 ***	−1.504 ***
	(0.266)	(0.189)	(0.332)
Employed (=1)	−0.270	−0.480 ***	−0.237
	(0.177)	(0.124)	(0.236)
Children (=1)	0.025	0.011	0.075
	(0.189)	(0.076)	(0.196)
Total assets	0.016	−0.098	−0.022
	(0.024)	(0.064)	(0.033)
Ln(GDP per capita)	−1.820	−0.675	−4.703
	(3.160)	(1.350)	(3.258)
Population density	−0.024	−0.007	−0.027
	(0.018)	(0.009)	(0.021)
Provincial FE	Yes	Yes	Yes
Year FE	Yes	Yes	Yes
Observations	2860	18,100	4233
R-squared	0.060	0.071	0.060

Note: *** *p* < 0.01, ** *p* < 0.05, * *p* < 0.1.

**Table 7:** Heterogeneity Effects by Educational Attainment.

Variable	Junior High or Below	High School Level	College or Above
Affordability	−0.623 ***	−0.560 ***	0.042
	(0.089)	(0.119)	(0.136)
Ln (age)	0.019 ***	−0.023 **	−0.014
	(0.006)	(0.009)	(0.011)
Gender (Male =1)	−1.001 ***	−0.457 ***	−0.247
	(0.087)	(0.126)	(0.165)
Married (=1)	−1.907 ***	−0.959 ***	−0.862 **
	(0.199)	(0.297)	(0.314)
Employed (=1)	−0.522 ***	−0.235	−0.521 **
	(0.120)	(0.179)	(0.194)
Children (=1)	0.047	−0.118	0.240
	(0.093)	(0.171)	(0.141)
Total assets	−0.061	−0.105	0.021
	(0.052)	(0.072)	(0.037)
Ln (GDP per capita)	−1.100	−3.628 *	−0.066
	(1.466)	(2.085)	(2.226)
Population density	−0.007	−0.021	−0.019
	(0.009)	(0.019)	(0.024)
Provincial FE	Yes	Yes	Yes
Year FE	Yes	Yes	Yes
Observations	18,758	3834	2601
R-squared	0.077	0.049	0.041

Note: *** *p* < 0.01, ** *p* < 0.05, * *p* < 0.1.

## Data Availability

The microdata for this analysis were obtained from the China Family Panel Studies (CFPS) for the waves 2016–2020, provided by Peking University (https://www.isss.pku.edu.cn/cfps/en/, accessed on 2 November 2023). Provincial macroeconomic indicators were sourced from the National Bureau of Statistics of China (http://www.stats.gov.cn/english/, accessed on 2 November 2023).
